# 3D tumour spheroids for the prediction of the effects of radiation and hyperthermia treatments

**DOI:** 10.1038/s41598-020-58569-4

**Published:** 2020-02-03

**Authors:** Sarah C. Brüningk, Ian Rivens, Carol Box, Uwe Oelfke, Gail ter Haar

**Affiliations:** Joint Department of Physics at The Institute of Cancer Research and The Royal Marsden NHS Foundation Trust, London, SM25NG UK

**Keywords:** Cancer models, Cell death

## Abstract

For multimodality therapies such as the combination of hyperthermia and radiation, quantification of biological effects is key for dose prescription and response prediction. Tumour spheroids have a microenvironment that more closely resembles that of tumours *in vivo* and may thus be a superior *in vitro* cancer model than monolayer cultures. Here, the response of tumour spheroids formed from two established human cancer cell lines (HCT116 and CAL27) to single and combination treatments of radiation (0–20 Gy), and hyperthermia at 47 °C (0–780 CEM_43_) has been evaluated. Response was analysed in terms of spheroid growth, cell viability and the distribution of live/dead cells. Time-lapse imaging was used to evaluate mechanisms of cell death and cell detachment. It was found that sensitivity to heat in spheroids was significantly less than that seen in monolayer cultures. Spheroids showed different patterns of shrinkage and regrowth when exposed to heat or radiation: heated spheroids shed dead cells within four days of heating and displayed faster growth post-exposure than samples that received radiation or no treatment. Irradiated spheroids maintained a dense structure and exhibited a longer growth delay than spheroids receiving hyperthermia or combination treatment at (thermal) doses that yielded equivalent levels of clonogenic cell survival. We suggest that, unlike radiation, which kills dividing cells, hyperthermia-induced cell death affects cells independent of their proliferation status. This induces microenvironmental changes that promote spheroid growth. In conclusion, 3D tumour spheroid growth studies reveal differences in response to heat and/or radiation that were not apparent in 2D clonogenic assays but that may significantly influence treatment efficacy.

## Introduction

Whilst monolayer cell cultures have underpinned decades of cancer research, they have been criticized for not being reliable predictors of treatment response *in vivo*^1,2^. The culture geometry of a flat, rigid substrate limits the number of neighbouring cells, resulting in reduced inter-cellular contact and communication compared with cells *in vivo*. Two dimensional (2D) culture conditions cause cells to stretch, increasing the surface area directly exposed to culture medium^3–5^. Cancer cells cultured in 2D are thus always provided with oxygen and nutrients. These are more restricted in the physiological microenvironment of a tumour where inefficient vasculature prevents their homogeneous supply. Despite these limitations, advantages such as low cost, high throughput, ease of application and control over microenvironmental factors have made monolayer cultures the standard technique for evaluation of cellular responses. However, there may be considerable inconsistencies between experimental results obtained *in vitro* in 2D culture and those observed *in vivo*.

One possible way of bridging this gap is to use three dimensional (3D) tumour spheroid cultures. Here, cells are grown as aggregates of single or multiple cell types, having 3D cell-cell contact, and proliferating in a more physiological geometry that stimulates production of extracellular matrix proteins and enhances inter-cellular communication^6,7^. Due to the 3D nature of spheroids, gradients of nutrients, oxygen and pH establish over time, better resembling the physiological microenvironment of solid tumours in which distance from blood vessels gives rise to these. Spheroids have a layered cellular structure with an outer proliferating, and inner quiescent zone, potentially with a necrotic core at their centre^4,8^. Although spheroids lack vasculature and cellular heterogeneity in terms of mutations and clonal evolution^3–5^, it has been shown that gene expression profiles differ in 2D and 3D cultured cells and, importantly, that 3D molecular fingerprints more closely resemble those of patient tumours than those seen in 2D^3,9–12^. When evaluating biological effects for the purpose of quantifying patient treatment efficacy, 3D spheroid assays may thus reflect the response of cells within a tumour better than those in 2D cultures. Increased resistance to heat or radiation (measured as clonogenic survival) of cells treated in 3D rather than 2D culture has been suggested^13–18^ potentially due to enhanced repair capability if cells are grown and treated in 3D. But it remains to be shown how this effect translates into spheroid growth response where cells remain in the spheroid microenvironment after treatment rather than being disaggregated and plated at (non-physiological) clonal densities. Although spheroid cultures had already been introduced in the 1970’s^19^, recent advances in high-throughput analysis using micro-well spheroid arrays and automated imaging now enable larger scale studies^20–23^.

The combination of radiotherapy (RT) with local hyperthermia (HT)^24–26^ using, for example, focused ultrasound^27–29^, may be promising for the treatment of radio-resistant tumours for which dose escalation is limited due to normal tissue toxicity. In order to maximise patient benefit, treatment planning needs to account for the biological effects of heat-induced radiosensitization. This is currently achieved using 2D clonogenic survival assays that provide the basis for the biologically weighted equivalent dose (BEQD) calculations^30,31^ that allow quantification of the effects of HT or RTHT treatments in terms of RT doses of equivalent biological effectiveness. Given the non-physiological cell microenvironment present when isolated cells are grown as monolayers, and an inability to account for differences in the dynamic cell death mechanisms induced^32–34^, the applicability of biologically weighted (thermal) dose prescription based on this assay alone is questionable. Analysis of 3D tumour spheroids can be used to test the applicability of BEQD calculations and provide a more accurate quantification of the effects of RT, HT and combinations thereof, to ultimately improve patient dose prescription.

Here, 3D spheroids from two human cancer cell lines were treated with combinations of high temperature HT (47 °C) and RT. The aim was to evaluate differences in spheroid growth dynamics following treatment, to test whether planning of combined RTHT treatments based on BEQD calculations relying purely on 2D clonogenic survival data is predictive of spheroid “response”. This was done by monitoring spheroid growth, cell viability and the spatial distribution of dead cells within spheroids. Results were compared with 2D clonogenic assays and 2D growth curves for a broad range of thermal (0–780 CEM_43_) and radiation doses (0–20 Gy).

## Results

### Analysis of response to thermal and radiation dose

#### Spheroid response to hyperthermia

**Figure 1:**
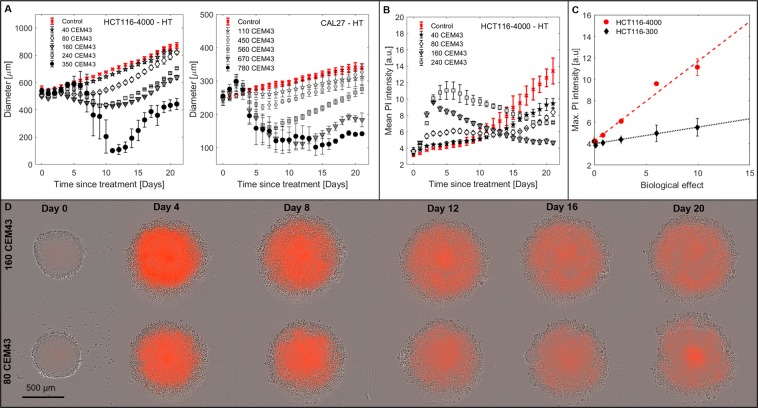
Spheroid response to HT. (**A**) Growth curves for HCT116-4000, and CAL27 spheroids following HT treatment 96 h after seeding. Some experimental data omitted to enhance readability. (**B**) Dynamic variation in mean PI staining intensity, indicative of cell death, for HCT116-4000 spheroids. (**C**) Peak mean PI intensity between days 0 to 7 within HCT116-300 and HCT116-4000 spheroids shows a linear increase (dashed lines, ${R}_{HCT116-300}^{2}\,=\,0.95$, ${R}_{HCT116-4000}^{2}\,=\,0.98$) with biological effect. (**D**) Phase contrast images overlaid with PI fluorescence snap-shots from time-lapse imaging of representative HCT116-4000 spheroids heated with 160 CEM_43_ or 80 CEM_43_. Spheroids showed intense PI staining (orange) throughout four days after treatment and a looser overall structure (relative to day 0) before cells were shed forming a corona around the remaining, growing spheroid. Means and standard errors of the mean of at least three independent repeat experiments are shown in A to C ($n\ge 3$).

HT treatment (Fig. 1A) resulted in a thermal dose-dependent growth delay. For high (350 CEM_43_ for HCT116, ≥560 CEM_43_ for CAL27) thermal doses, spheroid diameter initially (up to day 3 for CAL27 or day 6 for HCT116) continued to increase before significant shrinkage took place. At this point, spheroids disintegrated and reformed from at least one cluster of growing cells, as shown in Fig. 1D, and Supplementary Video 1. Variations in the disintegration time point between repeat experiments contributed to relatively large spheroid diameter error bars during this period (Fig. 1A).

Time-lapse imaging (see Fig. 1D for selected examples) showed that following HT, spheroids initially (days 1–4) displayed PI staining throughout and a more granular structure, indicating a loosening of cell packaging. Moreover, cell viability measurements on day 4 (Supplementary Fig. S1C) demonstrated that increasing thermal dose reduced spheroid viability, despite the diameter increase seen in Fig. 1A. Unlike later viability measurements (day 21), spheroid size was not a good indicator of post-HT cell viability on day 4 (Supplementary Fig. S1A,B), suggesting a change in cell density at this time point. Dead (PI positive) cells were shed during days 0–7 after heating and remained close to the regrowing spheroid body, forming a corona. Due to the U-shaped well format, outermost components of the corona (cells and cell fragments) were no longer within the focal plane of the central spheroid (fluorescent images only).

For both cell lines, heated spheroids that regrew had periods during which their growth rates were faster than comparably-sized, non-heated controls (see, for example, Fig. 1A HCT116 80 CEM_43_). Viability measurements (Supplementary Fig. S1A) demonstrated that, by day 21, spheroid diameter correlated with cell viability, suggesting that the observed accelerated growth rate was not an artefact of spheroid contouring uncertainty.

Dynamic variation in mean PI staining intensity is shown in Fig. 1B. Between days 1 and 15 after HT exposure, mean PI intensity was elevated for some thermal doses relative to untreated controls. Peak intensity observed between days 3–6 after treatment, correlated linearly with biological effect (Fig. 1C) for the thermal dose range 0 to 240 CEM_43_. Although HCT116-4000 spheroids showed no increase in growth delay between 160 and 240 CEM_43_ treatments (Fig. 1A), PI staining intensity was stronger in the samples receiving the higher thermal dose (Fig. 1B) indicating greater cell kill.

**Figure 2:**
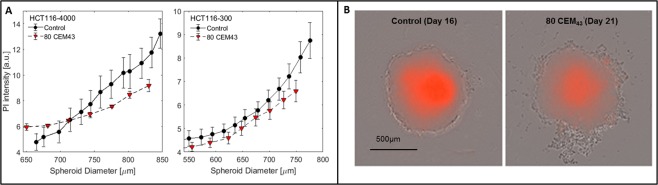
Comparison of heated and unheated spheroids at the same diameter. (**A**) Comparison of mean PI intensity in untreated (Control) and regrowing 80 CEM_43_ treated HCT116-4000 and HCT116-300 spheroids (days 14–21) as a function of diameter. (**B**) Phase contrast and overlaid PI fluorescence images of comparably sized examples of an untreated and a heated HCT116-4000 spheroid demonstrating that heated samples had fewer necrotic, PI stained, cells at their core.

Figure 2A shows a comparison of mean PI staining intensity in untreated controls and regrowing HCT116 spheroids (days 8 to 21) after treatment with 80 CEM_43_ as a function of spheroid diameter. PI staining in regrowing heated samples was generally less intense than in similarly sized controls (Fig. 2B). This effect was more pronounced in larger spheroids (HCT116-4000). Live fluorescent microscopy imaging revealed that the lower PI staining in regrowing heated samples was due to a smaller necrotic core than in comparably sized untreated controls (Fig. 2B), suggesting reoxygenation of central cells following HT.

#### Spheroid response to radiation

**Figure 3:**
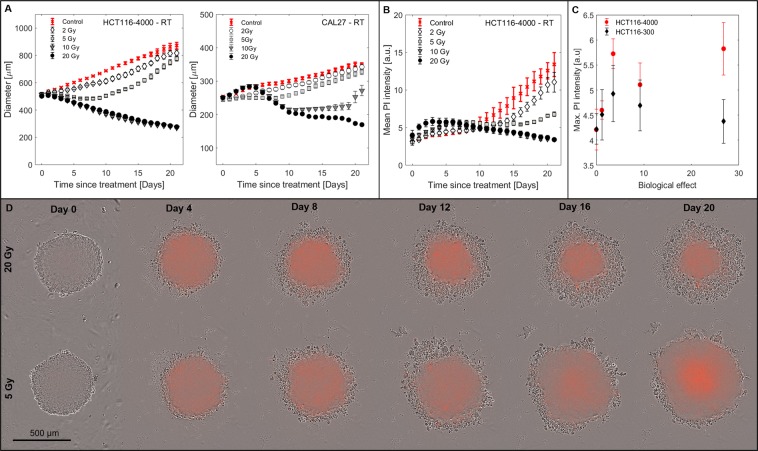
Spheroid response to RT. (**A**) Growth curves for HCT116-4000 and CAL27 spheroids following RT treatment 96 h after seeding. (**B**) Mean PI staining intensity as a function of time after irradiation for HCT116-4000 spheroids. (**C**) Peak mean PI intensity measured after RT (days 0–7) for HCT116-300 and HCT116-4000 spheroids plotted as a function of the biological effect showed poor linear correlation (${R}_{HCT116-300}^{2}\,=\,0.02$, ${R}_{HCT116-4000}^{2}\,=\,0.48$). (**D**) Phase contrast images overlaid with PI fluorescence snap-shots from time-lapse imaging of representative examples of HCT116-4000 spheroids irradiated with 20 Gy or 5 Gy. Irradiated samples showed faint PI staining throughout and retained a dense structure. Cells were continuously shed from the outer spheroid layers until regrowth occurred. Means and standard errors of the mean of at least three independent repeat experiments are shown in A to C ($n\ge 3$).

Figure 3A shows growth curves for HCT116-4000 and CAL27 spheroids measured as a function of radiation dose delivered, 96 h after seeding. Although some irradiated spheroids initially continued to grow at the same rate as untreated controls, all showed a reduction in growth rate between days 1 and 5. This resulted in a sustained dose-dependent growth delay with higher doses (see, for example, 20 Gy) showing no regrowth during the 21 day observation period. Cell viability measurements on day 4 (Supplementary Fig. S1C) showed no significant dose-dependence, indicating that cells initially remained metabolically active. By day 21 viability was reduced in the expected dose-dependent manner (Supplementary Fig. S1A). Time-lapse imaging (Fig. 3D, Supplementary Video 2) showed that irradiated spheroids retained a dense structure with dead cells detaching from the outer cell layers, resulting in gradual shrinkage from the outside inwards.

Dynamic variation in mean PI staining intensity was assessed for HCT116 spheroids (Fig. 3B). An increase in PI staining relative to controls was observed for days 0–7 after RT. This was, however, significantly less intense than observed for HT treatments. The peak in mean PI intensity (day 0–7) showed poor linear correlation with biological effect (Fig. 3C). PI intensity then decreased until the formation of a necrotic core resulted in signal increase towards the end of the observation period for regrowing samples (2 and 5 Gy). Samples irradiated with 10 and 20 Gy continuously shrank, preventing central necrosis, and resulting in a decrease in PI intensity due its dependence on the spheroid volume captured in the focal plane upon imaging.

#### Spheroid response to combinations of radiation and hyperthermia

**Figure 4:**
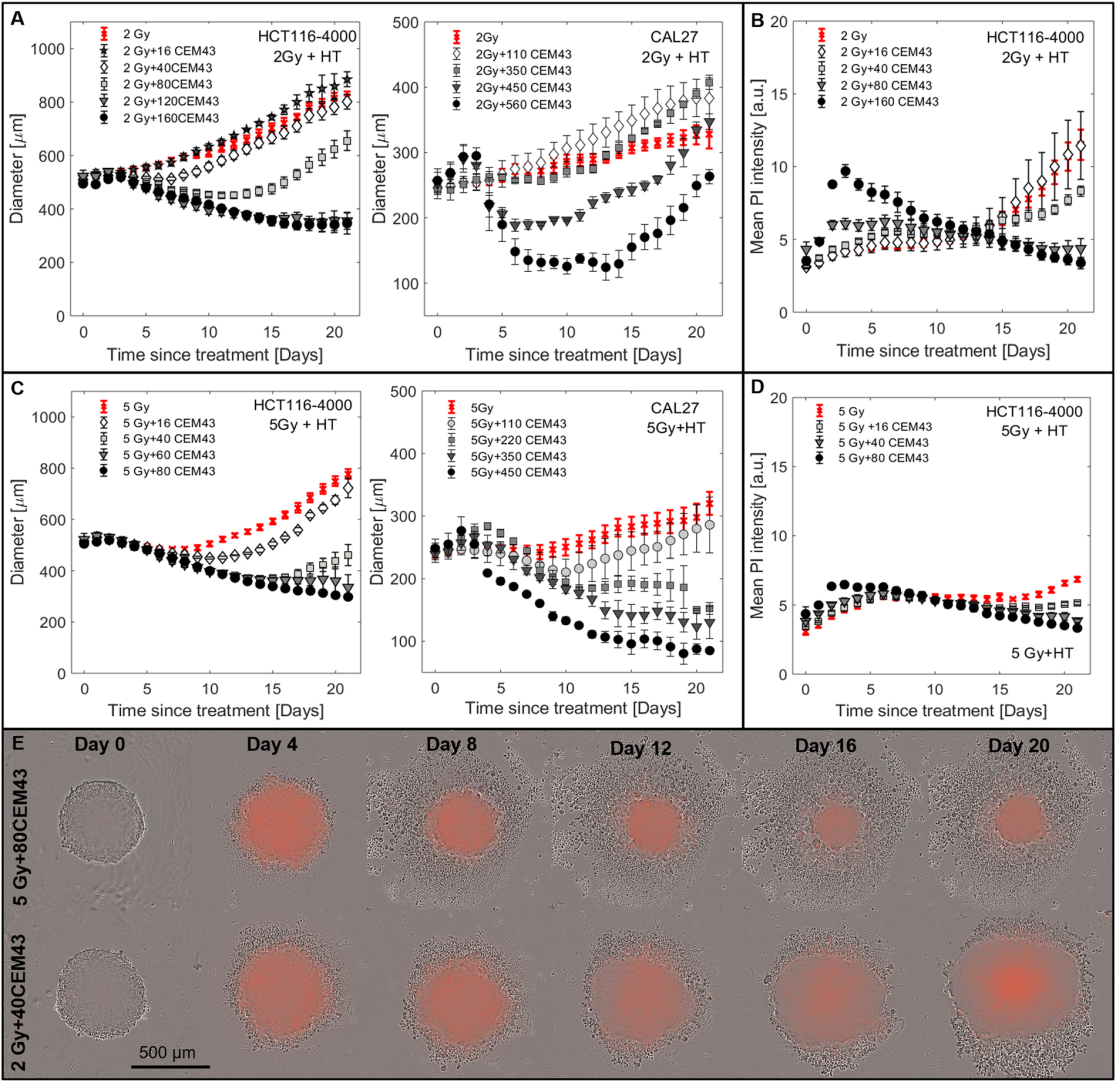
Spheroid response to RTHT. (**A**,**C**) Growth curves for HCT116-4000 and CAL27 spheroids following RTHT treatment 96 h after seeding. Combinations of HT (16 to 560 CEM_43_) with 2 Gy (**A**) or 5 Gy (**C**) are shown. (**B**,**D**) Mean PI staining intensity as a function of time after RTHT exposure (HCT116-4000 spheroids). (**E**) Phase contrast images overlaid with PI fluorescence snap-shots from time-lapse imaging of representative HCT116-4000 spheroids treated with 5 Gy + 80 CEM_43_ or 2 Gy + 40 CEM_43_. Means and standard errors of the mean of at least three independent repeat experiments are shown in A to D ($n\ge 3$).

Spheroid growth following combined RTHT (Fig. 4A,C, Supplementary Videos 3 and 4) shared characteristics of either RT or HT alone, with increasing thermal or radiation dose, both delaying spheroid growth. Similar to HT treatments, RTHT treated spheroids could grow faster than spheroids receiving only the relevant RT dose. This was particularly evident in CAL27 and HCT116 spheroids treated with a combination of 2 Gy + HT (Fig. 4A). Here, RTHT (for example 2 Gy + 16 CEM_43_ for HCT116, 2 Gy + 110 CEM_43_ for CAL27) treated spheroids rapidly (by day 5) exceeded the size of those receiving 2 Gy alone. Treatments combining 5 Gy RT with HT also led to growth rate increase (for example HCT116 5 Gy + 16 CEM_43_), however, within 21 days the spheroid size remained less than that of controls receiving 5 Gy alone, indicating that the additional fixed thermal dose delayed growth more when combined with higher radiation doses.

PI intensity within one week of treatment appeared to be driven by the thermal, rather than radiation, dose delivered (Fig. 4B,D). After RTHT, time-lapse imaging (Fig. 4E, Supplementary Videos 3 and 4) showed that, morphologically, growth response displayed characteristics of both RT and HT alone: thermal doses determined the amount of PI staining and cell shedding within one week of treatment (Supplementary Video 3), but spheroids also shrank continuously from the outside in for combinations with 5 Gy RT (Supplementary Video 4, Fig. 4C).

#### Monolayer growth curves

**Figure 5:**
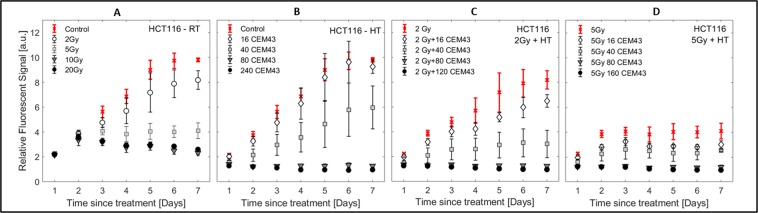
Monolayer growth curves. Resazurin assay performed on HCT116 cells for a subset of RT (**A**), HT (**B**) and RTHT (**C**,**D**) treatments. In all graphs, means and standard errors of the mean of at least three independent repeat experiments are shown ($n\ge 3$).

Study of growth of 2D monolayers (HCT116 cells Fig. 5, CAL27 Supplementary Fig. S2A) was limited to one week as the cell populations reached confluence. Following RT (Fig. 5A) cell number initially continued to grow (days 1, 2) before: continuously decreasing (10 and 20 Gy), plateauing (5 Gy), or showing slight growth delay relative to untreated controls (2 Gy). HT (Fig. 5B) resulted in an early (day 1–2) thermal dose dependent change in cell number relative to untreated controls. HCT116 cells either continued to proliferate (thermal doses <80 CEM_43_), or remained at a constant low number. Combination treatments (Fig. 5C,D) showed characteristics of both, RT and HT, responses: 2 Gy + 16 CEM_43_ caused slight growth delay, whilst 2 Gy + 80 or 120 CEM_43_ inhibited cell division. However, with 5 Gy, additional heating had a lesser effect since radiation alone inhibited cell division.

### Comparison of isotoxic treatments based on clonogenic survival

**Figure 6:**
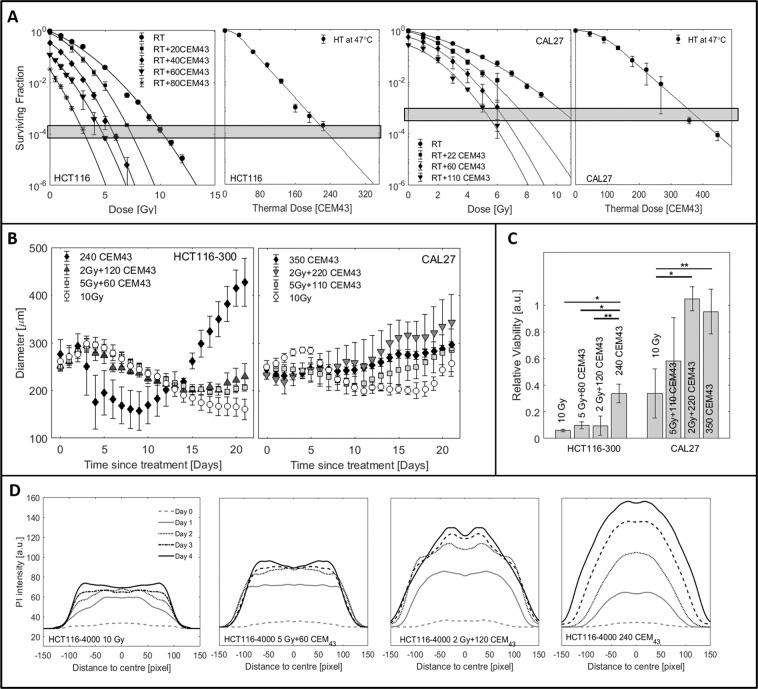
Comparison of growth responses after isoeffective treatments having the same 2D clonogenic survival fraction, delivered by either RT, HT or a combination thereof. (**A**) Clonogenic survival (mean values and standard deviations of at least three repeat experiments ($n\ge 3$)) of HCT116 and CAL27 cells as a function of radiation or thermal dose. BEQD levels seen for 10 Gy RT are indicated as horizontal boxes. (**B**) Growth curves for HCT116-300 and CAL27 spheroids. Mean values and standard errors of at least three independent experiments are shown. (**C**) Cell viability measured by Cell-Titer-Glo3D 21 days after treatment of the samples shown in B. *p < 0.01, **p < 0.05. (**D**) Spatial distribution of PI staining within representative examples of treated HCT116-4000 spheroids. The x-axis indicates the distance in pixels from the spheroid centre of mass.

To evaluate the applicability of BEQD based on clonogenic survival (Fig. 6A and^35^), HCT116 and CAL27 spheroids were treated with RT, HT and RTHT at the same BEQD level (equivalent to 10 Gy). Spheroid growth, cell viability, spheroid morphology and distributions of live and dead cells were compared at these isoeffective doses (Fig. 6, Supplementary Videos 1–4). Despite comparable levels of surviving cells in monolayers (Fig. 6A), spheroid growth differed with treatment regimes used (Fig. 6B). For both cell lines, there was little (CAL27), or no (HCT116), significant regrowth within 21 days after 10 Gy RT, whereas both heated (240 CEM_43_ for HCT116, 350 CEM_43_ for CAL27), and RTHT treated samples began to regrow within this time. For HCT116 spheroids (Fig. 6B, left) delay until regrowth decreased with thermal dose contributing an increasing proportion of the treatment, and by day 21 HT treated spheroids were significantly ($p\, < \,0.01$) larger than RT and RTHT treated samples. This trend was not as clear for CAL27 spheroids, with no statistically significant differences (all $p\, > \,0.05$) in spheroid diameter on day 21 between any of the treatment regimes due to large variations in spheroid diameter after RT, and RTHT (Fig. 6B, right). Cell viability assays on day 21 indicated significant treatment regime differences (Fig. 6C): 10 Gy RT only treated spheroids were significantly less viable than samples receiving the same BEQD delivered by HT alone (both cell lines, $p\, < \,0.05$), or by 2 Gy + HT (only CAL27, $p\, < \,0.01$). The spatial distribution of PI staining of HCT116-4000 spheroids on days 0–4 (Fig. 6D) exposed to heating alone showed the highest levels of staining throughout, with centralised peak enhancement and gradual widening of the stained area over time. Irradiated samples generally showed less staining. By day 4 this was enhanced towards the spheroid edges, indicating that the outer (proliferating) cell layers were predominantly affected by RT-induced cell death. With increasing thermal dose contribution to combination treatments, PI staining intensified and central cell populations were more affected. PI staining increased within 4 days after treatment and the stained area increased over time (2 Gy + HT).

**Figure 7:**
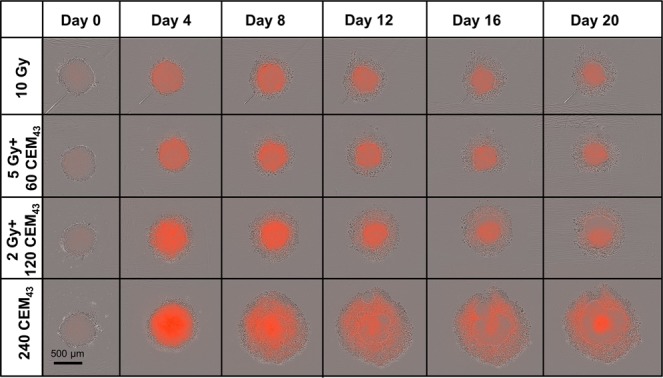
Comparison of growth response after isoeffective treatments using time-lapse imaging. Phase contrast images overlaid with PI fluorescence snap-shots from time-lapse imaging of representative HCT116-4000 spheroids treated with isoeffective doses of: 10 Gy (top), 5 Gy + 60 CEM_43_ (2nd row), 2 Gy + 120 CEM_43_ (3rd row), or 240 CEM_43_. Despite comparable levels of 2D clonogenic survival, the dynamics of cell death and cell detachment were significantly different between the treatment regimes, resulting in considerable differences in spheroid growth delay.

Time-lapse imaging (Fig. 7) revealed significantly different cell death and detachment dynamics for different treatment regimes despite isoeffective doses. Heated spheroids began to disintegrate within one week of treatment, shedding dead cells that formed a corona around the (still growing) central spheroid. In contrast, irradiated spheroids gradually shrank from the outside inwards, always maintaining a dense structure, with dead cells being shed continuously. Spheroids receiving combination treatments displayed a mixture of the morphologies of the single treatments. Samples receiving 2 Gy + 120 CEM_43_ displayed more intense PI staining on day 4, and slightly more shed cells relative to those receiving 10 Gy or 5 Gy + 60 CEM_43_ before regrowth could be observed (around day 16). In contrast, 10 Gy irradiated spheroids gradually shrank from the outside in, always maintaining a dense structure with relatively few dead cells being shed, and forming a smaller corona than for HT treatments.

## Discussion

In this study we have compared cellular responses to radiation, hyperthermia, or combinations of the two treatments when tumour cells were cultured as monolayers or as 3D tumour spheroids. A range of assays was used to characterise the dynamic response of the treated cell populations.

An increased heat resistance was observed in 3D cultured cells relative to monolayer response. For both HCT116 and CAL27 cells, HT alone up to 350 CEM_43_ (HCT116) or 780 CEM_43_ (CAL27) achieved spheroid disintegration but failed to provide spheroid growth control within the 21 day observation period (Fig. 1A). These thermal doses correspond to very low 2D clonogenic survival levels (${S}_{CAL27}(780\,CE{M}_{43})\, < \,{10}^{-7}$, ${S}_{HCT116}(350\,CE{M}_{43})\, < \,{10}^{-6}$). Moreover, HCT116 monolayers did not grow following thermal doses ≥$80\,CE{M}_{43}$ (Fig. 5B), whereas spheroids regrew after treatment with higher thermal doses (Fig. 1A, left). Similar results were obtained for CAL27 cells (Supplementary Fig. S2), suggesting that 2D cultured cells were significantly more heat-sensitive than 3D cultures. This is in agreement with previous studies comparing the proportion of necrotic and apoptotic cells^17^, or clonogenic survival^13^ in heated 2D and 3D cultures. The results presented here additionally demonstrate spheroid (re)growth following HT, rather than cell death or clonogenic potential alone. Differences in initial cell number between 2D and 3D cultures (2000 for 2D, ~4800 for HCT116-300 spheroids) cannot explain spheroid regrowth at the given 2D survival levels, meaning that heat response is influenced by factors specific to the 3D culture, such as the cellular microenvironment or enhanced cell-cell contact. Durand *et al*.^13^ reported a build up of thermotolerance at temperatures >43 °C in cells heated as spheroids relative to those heated in monolayer cultures. This agrees with our observation in HCT116 spheroids, where growth delay did not increase following HT between ≈160–240 CEM_43_. For CAL27 spheroids, however, an increase in growth delay as a function of thermal dose was observed. This may suggest an increase in heat tolerance due to a “contact effect”, as described previously for irradiated spheroids^36^. This may be due to preferential killing of proliferating differentiated, rather than cancer stem cells, as previously suggested for HCT116 cells^37^. Another explanation could be differences in the expression of heat shock proteins (HSPs), such as the molecular chaperone HSP70, in monolayer and spheroid cultures as suggested by experiments of Song *et al*.^17^. Since the responses observed were cell-line-specific, more experimental analysis is needed to elucidate the underlying mechanism. Microenvironmental factors such as oxygenation or pH were not expected to increase spheroid heat tolerance: HCT116-300 and CAL27 spheroids had no regions of severe hypoxia (Supplementary Fig. S3) on treatment day. Spheroids have been shown to be potentially more acidic at their core^38^, but increased extracellular pH is associated with increased cellular heat sensitivity^39–41^.

Moreover, this analysis suggests that cell death dynamics impact on spheroid growth response. Cells in monolayer cultures are uniformly exposed to nutrients and oxygen, grow exponentially, and can detach at any time from the substrate in the event of cell death. In contrast, as for tumours, cell populations within a spheroid are heterogeneous and evolve dynamically after treatment as a consequence of continuing cell death and proliferation of surviving cells. The spheroid microenvironment (constituting gradients of oxygen, nutrients and waste products) constantly changes as its size varies and this impacts on cell proliferation, and on the ultimate efficacy of any treatment. Differences in dynamic cell detachment from spheroids induced by RT or HT were found to influence population growth response and viability.

In agreement with observations in other cancer cell lines^42,43^, HCT116 and CAL27 spheroids maintained a compact structure after irradiation and shrinkage occurred from outer cell layers (see Fig. 3D, Supplementary Video 2). This resulted in spheroid growth control for doses of 10 Gy (only HCT116) and 20 Gy. Radiation-induced cell death was thus mostly restricted to the outermost spheroid cell layers. This suggests that quiescent cells first re-entered the cell cycle before cell killing became effective (as previously suggested^42,43^). Maximum PI staining did not correlate with biological effect in the case of RT. This is in agreement with the suggestion that delayed cell death resulting from aberrant mitosis provides a major contribution to radiation-induced cell killing^34^ and therefore cells continue to die over time after RT. In contrast, heated spheroids shed dead cells from the main spheroid body, or completely disintegrated, within a week of treatment, resulting in an apparent transient increase in spheroid diameter due less compact cell packaging, followed by rapid shrinkage (see Fig. 1D, Supplementary Video 1). This observation is in agreement with previous studies^44^ that reported a loosening of the spheroid structure and loss of cell adhesion after HT. We also observed no enhanced PI staining at HCT116-4000 spheroid edges after HT (Fig. 6D) and suggest that HT-induced cell death is independent of the spatial location within the spheroid, so that both proliferating and quiescent populations were killed simultaneously. The observed increase in PI staining at the spheroid core directly after HT may be the result of more acidic pH towards the centre affecting both cell survival and PI staining intensity. PI staining peaked approximately four days after HT. The maximum PI intensity also correlated with biological effect (see Fig. 1C), and at this time point (day 4) cell viability reduced as a function of thermal dose. Both observations indicate that following HT all cells died within a few days of treatment via cellular necrosis or apoptosis induced at 47 °C as reported previously^17,34,45^.

In both cell lines tested, heated spheroids grew faster at some points after treatment than did unheated controls at any point between 0 and 21 days (Figs. 1A, 4A and S2B panel 2). This is most obvious for combinations of 2 Gy radiation with low thermal doses (Fig. 4A), where heated spheroids grew larger and were more viable than those receiving radiation alone. This was an unexpected finding. It could be speculated that the selective killing of slow growing subpopulations by HT leads to a net increase in spheroid growth rate. However, growth rate depends on microenvironmental and cell density changes due to the death and clearance of cells, enabling reoxygenation and a fresh supply of nutrients to the core of the spheroid. This was confirmed by the observation that three weeks after treatment with 80 CEM_43_ the average PI intensity of HCT116 spheroids was significantly lower than that of controls of similar size (see Fig. 2A). This effect was more pronounced in larger, more hypoxic HCT116-4000 spheroids. We suggest that this may be due a smaller necrotic core as confirmed by time-lapse imaging (see Fig. 2B), but it should be noted that PI fluorescence intensity may be influenced by the physicochemical composition of the cellular microenvironment. Thus, confirmation of either of these hypotheses and further investigation, for example, using histological analysis, is required. Although an increase in growth rate of heated spheroids may seem to contradict the use of hyperthermia, it should be stressed that the *in vitro* spheroid culture does not account for other potential beneficial physiological effects of this treatment *in vivo*, such as immune stimulation^46,47^. Moreover, re-oxygenation and active proliferation of previously quiescent cell populations sensitises these to fractionated radiation treatments and it would thus be essential to further investigate the efficacy of fractionated treatments which could indeed show a further benefit of combined RTHT treatments despite an enhanced growth of spheroids following a single treatment fraction.

In summary, it is suggested that clonogenic survival alone may be insufficient to calculate biologically equivalent doses. Clonogenic survival and 2D growth data provide a valid indication of a cell line’s intrinsic treatment sensitivity: of the two cell lines studied here, HCT116 cells were more sensitive to both RT and HT than CAL27 cells in 2D survival and growth assays (steeper clonogenic survival curve, growth control achieved at lower thermal/radiation doses). Similarly, in 3D assays HCT116 had reduced growth delay and cell viability for given thermal or radiation dose, as seen, for example, in the 10 Gy and 240 CEM_43_ data. Since 2D responses are not recapitulated in 3D cultures they are therefore unlikely to be predictive of response *in vivo*. Specifically, spheroid growth and viability differed significantly after treatment with equivalent BEQD levels of RT, HT and RTHT calculated from clonogenic survival data (see Fig. 6B,C). Our work demonstrated that calculations based on clonogenic survival alone could overestimate HT (and RTHT) treatment efficacy (Fig. 6B,C), if effects are assessed relative to RT as previously suggested^30,31,48,49^.

It is expected that RT and HT responses would be influenced by the cellular microenvironment: hypoxic cells are more resistant to RT^32,50^, and more sensitive (or of equal) response to HT^51,52^ with potential additional heat sensitizing effect due to a more acidic pH at the spheroid core^39,40^. Yet, RT in HCT116-4000 spheroids which had a distinct hypoxic core on treatment day resulted in longer growth delay, smaller spheroid volume and reduced cell viability three weeks after treatment than HT. Additional factors, such as differential effects arising from treatment specific cell death dynamics and microenvironmental changes induced, should be accounted for in the calculation of BEQD for multimodality therapies. Tumour spheroid models involve microenvironmental features and gradients of cell proliferation that could enable more accurate BEQD calculations. However, ambiguities in the definition of treatment response endpoints make it difficult to define such an alternative BEQD concept based on spheroid assays. With the exception of clonogenic survival, current response metrics are dependent on the evaluation time point (such as cell viability, live/dead assays), the size of the treated population (such as spheroid control probabilities), and/or the current growth rate (time to regrowth). More sophisticated, spheroid data-based BEQD calculations that account for the dynamic difference in the spatial patterns of cell death induced by RT and HT may provide a way forward and should be further investigated. For single modality treatments, clonogenic assays may provide a good indication of treatment response, since the underlying cell death dynamics are comparable. However, if treatment modalities are combined, differing cell death patterns and the resulting growth responses should be accounted for during treatment planning.

Limitations of this study were the use of a single treatment fraction (as discussed above), an evaluation at a fixed temperature of 47 °C, and the use of a single cell type in our 3D model. Important intercellular interactions present in tumours *in vivo*, such as cancer-stromal cell interaction, were not represented in our chosen *in vitro* model. Further evaluation of treatment response should be conducted in more complex, co-culture models comprising stromal, endothelial and immune cells in addition to tumour cells, to more accurately recapitulate the tumour microenvironment. Standard clinical care currently comprises fractionated therapy with temperatures ranging between 39–45 °C^53–55^. In order to relate our *in vitro* results to these standard treatment regimes, it should be noted that the concept of thermal dose has been validated for temperatures up to 50 °C^35,56^ and that the proportion of cells killed by hyperthermia could thus be transferable to different temperature regimes. However, we demonstrated that the spheroid cell culture system could influence the cells’ heat-sensitivity. The applicability of the thermal dose concept in its current form based on clonogenic cell survival should therefore be further investigated. Previous studies^45,57^ also suggest that the mechanism of heat-induced cell death (apoptosis, necroptosis, or necrosis) may depend on temperature but threshold temperatures for the onset of heat-induced necrosis may vary significantly depending on cell type. Since the treatment duration and temperature range was limited in our study due to the use of a thermal cycler, we encourage further analysis of dynamic spheroid response in a temperature dependent manner. It also remains to be shown how temperature-dependent, heat-induced physiological changes, such as enhanced or decreased perfusion or immunogenic effects, further influence the reported cell death dynamics and tumour response. After optimization of fractionation and treatment scheduling for RTHT treatments in spheroids, *in vivo* tests need to quantify the influence of these physiological effects as a function of thermal dose, heating temperature, and treatment fractionation.

## Methods

### Cell lines and culture conditions

Two human cancer cell lines, HCT116 (colorectal carcinoma), and CAL27 (squamous cell carcinoma) were obtained from the American Type Culture Collection. Screening for mycoplasma contamination was performed by polymerase chain reaction (by Surrey Diagnostics, Cranleigh, UK) and both cell lines were authenticated by short tandem repeat analysis using a Gene Print 10.0 kit (Promega, Madison, USA). Cells were cultured in Dulbecco’s Modified Eagle’s Medium (Gibco Life Technologies Ltd. Paisley, UK) supplemented with 10% foetal bovine serum (PAN Biotech GmbH, Aidenbach, Germany). Cell monolayers were grown in standard tissue culture flasks in a humidified incubator with 5% CO_2_ at 37 °C. Cells were passaged twice per week using a trypsin like enzyme, TrypLE^®^ (Gibco Life Technologies Ltd), for gentle detachment of the cells. For experiments, cells were used in exponential growth phase having undergone fewer than 10 consecutive passages.

For spheroid formation a fixed number of cells (4000 for CAL27, 4000 or 300 for HCT116, denominated as HCT116-300 and HCT116-4000) was seeded in 200 *μl* of complete growth medium in round bottomed, ultra-low attachment 96-well plates (ULA plates #7007, Corning B.V. Life Sciences, Amsterdam, Netherlands) and incubated for 96 h to allow time for gradients in oxygen and nutrients to establish^2^. After 96 h, 4000 CAL27, or 300 HCT116 cells produced a spheroid of approximately 300 *μm* diameter, with no signs of hypoxia or necrosis on treatment day. HCT116-4000 (the seeding number for CAL27) yielded much larger spheroids (approximately 550 *μm* diameter after 96 h) that possessed a hypoxic, yet not necrotic, core on treatment day (Supplementary Fig. S3). During the observation period, medium was renewed every three days by removing 100 *μl*, and adding back the same volume of fresh complete growth medium.

### Treatment procedure and dosing

For treatment, spheroids were transferred from ULA plates in 60 *μl* of complete growth medium/well into thin walled 200 *μl* PCR tubes (VWR Int., Lutterworth, UK) using wide bore pipette tips (Alpha Laboratories, Eastleigh, UK) to minimise spheroid damage. For 2D cultures, cell suspensions ($5\cdot {10}^{6}\,cells$/*ml*) were prepared and transferred in 60 *μl* aliquots into PCR tubes. Radiation and thermal exposures were performed as previously described^35^. A small animal radiation research platform (SARRP, X-Strahl, Camberley, UK) provided doses up to 20 Gy (220 kVp, 13 mA, beam hardened using 0.14 mm Cu filtering). Heating was delivered in a Tetrad thermal cycler (Bio-Rad Laboratories Inc., Berkeley, USA). In the following, all heat treatments are quantified as “thermal dose” in units of CEM_43_ (computed equivalent minutes at 43 °C) which is defined as the heating time at 43 °C yielding an equal clonogenic surviving fraction as the treatment delivered at a different temperature and duration. Here, thermal dose was calculated using the AlphaR model as previously described^35^, rather than the Sapareto formula^58^, to account for differences in thermal dose calculation for each cell line. For all experiments heating temperature was 47 °C and thermal doses up to 780 CEM_43_ were delivered. The choice of 47 °C was motivated by the application of combination treatments of focused ultrasound-mediated partial tumour ablations with RT^29^ where heat diffusion from the ablated volume would sensitize tumour cells outside the ablation zone. In such a case, heating temperatures of 45–50 °C will be delivered for minutes. This time-temperature combination was also a compromise between treatment time and contribution of heating ramp up/down gradients to the total thermal dose in the thermal cycler. For all combination treatments, heat was delivered within 30 min of irradiation and tubes were kept on ice before, between and after treatments.

Treated spheroids were transferred to fresh ULA plates and medium was topped up to 200 *μl* using culture medium supplemented with 1% mix of penicillin/streptomycin solution (P4333, Sigma Aldrich Ltd.) and 23 *μg*/*ml* amphotericin B (A2942, Sigma Aldrich Ltd.) to minimize microbial contamination risk resulting from transferring spheroids between vessels. Optionally, propidium iodide (PI, Sigma Aldrich Ltd.) was added at a concentration of 5 *μ*g/ml to the culture medium to allow visualization of dead cells^59–61^. PI (5 *μ*g/ml) was also added to growth media used to replenish 3D cultures ensuring that it was never limiting and could freely diffuse into spheroids, irrespective of their diameter or how tightly or loosely packed the cells were. It was confirmed that the addition of PI did not influence spheroid growth (Fig. S4), and that the formation of a necrotic core was observed in both PI stained live images and in H&E stained spheroid sections (Supplementary Material). Plates were incubated in a humidified incubator (5% CO_2_) at 37 °C for up to three weeks. For each treatment, a minimum of three independent repeat experiments each using four to six spheroids per condition was performed ($n\ge 3$).

Besides analysis in a thermal and radiation dose dependent manner (0–780 CEM_43_, 0–20 Gy, 2 Gy or 5 Gy with HT (0–550 CEM_43_)), spheroid response was also evaluated for RT and thermal doses which gave clonogenic surviving fractions comparable to those from 10 Gy RT alone, to test whether BEQD-based on clonogenic survival yielded similar spheroid response. A dose of 10 Gy was chosen since it resulted in spheroid growth control (HCT116) or long growth delay (CAL27) and a change in treatment efficacy from HT relative to RT, would therefore be clearly identifiable within the observation period. For CAL27 spheroids a survival level of ≈6 · 10^−4^ (10 Gy, 2 Gy + 220 CEM_43_, 5 Gy + 110 CEM_43_, 350 CEM_43_) was chosen, for HCT116 this level was ≈1 · 10^−4^ (10 Gy, 2 Gy + 120 CEM_43_, 5 Gy + 60 CEM_43_, 240 CEM_43_). All clonogenic surviving fractions(*S*) and biological effect levels (−*ln*(*S*)) were calculated using the AlphaR model and clonogenic cell survival data for HCT116 and CAL27 cells as described previously^35^.

### Response evaluation

#### Spheroid diameter and mean PI intensity

Spheroid growth was assessed three times per week over a period of three weeks after treatment using bright field microscopy images acquired on a Celigo™imaging cytometer (Nexcelom Biosciences LLC, Lawrence, USA), at a resolution of 1 *μm*/pixel. Spheroid contours were automatically segmented using an implementation of a deep learning, convolution neural network (CNN). This CNN was adapted from a conventional U-net architecture^62^ with four levels and 128 first level feature layers implemented in TensorFlow (https://www.tensorflow.org/) with Keras (https://keras.io/), and used learned feature compression rather than a static pooling compression. The OpenCV2 (https://docs.opencv.org) library in Python was used to identify connected pixels in the geometrical neighbourhood of a pixel that posed a high probability of being part of a spheroid body. The CNN was trained on over 3000 manually segmented contours. Training was performed in 120 epochs on a GTX1080 Ti GPU within six hours and reached a dice similarity coefficient of over 0.92. Automated segmentation ensured consistent contouring and minimised random variations where contours were difficult to identify.

The spheroid diameter was calculated from the average of 36 diameters through the centre of mass of the segmented area (every five degrees). Pooling, averaging and visualisation of the data was performed in MATLAB (MathWorks^®^, version 2017a, USA). Here, the mean diameter (and standard deviation) of all spheroids within each repeat experiment was averaged for each scan time. These data points were linearly interpolated to obtain spheroid diameter values at fixed time points post treatment (0 to 21 days in daily increments). The values obtained were averaged over independent repeats ($n\ge 3$) and growth curves were plotted using mean values and standard errors of the mean.

Fluorescence images were captured on a Celigo™ using 535/617 nm excitation/emission wavelengths and a constant exposure duration of 48 ms. Fluorescent signals were analysed in MATLAB as overall mean intensities within each image. Data were averaged as described above for the diameter measurements. Maximum (mean) PI intensities within one week post treatment were evaluated as a function of biological effect (which refers to the negative logarithm of clonogenic survival for this (thermal) dose^35^) to analyse the proportion of treatment-induced cell death. For CAL27 spheroids the presence of necrotic patches even in newly formed, untreated controls (Supplementary Fig. S3) hindered quantification of PI. Staining intensity is therefore only shown for HCT116 spheroids. The measured intensity of this DNA intercalating dye may, however, vary depending on cell cycle stage (due to DNA content), the physicochemical properties of the microenvironment (pH, presence of different cations), and the depth of the labelled cell within the spheroid (diffusion into the spheroid, attenuation of fluorescent signal). PI measurements were thus only compared between treatments and were not used to quantify the observed qualitative distribution of dead cells in terms of cell viability.

#### Time-lapse imaging

Time-lapse imaging of spheroids was performed on an IncuCyte S3 (Sartorius AG, Göttingen, Germany) live-cell analysis system. PI fluorescence and high definition phase contrast images were automatically acquired every four hours for 21 days after treatment at 40X magnification. For quantification of spatial distribution of PI intensity, line profiles were taken every 5 degrees through the centre of mass of the Gaussian filtered fluorescent image of representative examples of spheroids in MATLAB. The line profiles obtained were aligned centrally and the average line profile was calculated.

#### Cell viability testing

Cell viability in spheroids was assessed using the Cell-Titer-Glo™3D reagent (Promega) following the manufacturer’s protocol. Briefly, 100 *μl* of culture medium was replaced with an equal volume of room temperature Cell-Titer-Glo™3D reagent. After mixing followed by 30 min incubation, under agitation in the dark, the contents of each well were transferred to a white, flat bottom 96-well plate and the luminescent signal in each well was measured using a spectrophotometer multi-plate reader (FluoStar, Omega, BMG Labtech, Ortenberg, Germany). Cell viability was assessed at the end of volume growth monitoring (at 21 days) or 4 days after treatment for a subset of thermal/radiation doses. Viability is reported relative to untreated controls, and all data are the means and standard deviations of at least three experiments ($n\ge 3$). Statistical analysis was performed in GraphPad Prism using an unpaired t-test.

#### 2D assays

Clonogenic survival was evaluated using the colony forming assay described previously^35^. The resazurin assay was used to measure 2D growth curves over one week. On day 0, 200 *μl* of cell suspension containing 2000 (treated) cells were plated in flat bottom 96-well plates. For each condition, six replicate wells and a total of six well-plates were used. Plates were incubated at 37 °C in a CO_2_ incubator and one was removed every 24 h for viability testing. The plate was subjected to a resazurin assay, as follows: 100 *μl* of culture medium per well was removed, and replaced with 20 *μl* resazurin solution (0.15 mg/ml (Sigma Aldrich Ltd.) in Dulbecco’s PBS, filter sterilized through a 0.2 *μm* membrane filter). After incubation for 4 h at 37 °C the fluorescent signal at an excitation/emission wavelength of 560/590 nm was measured using a multi-plate spectrophotometer (FluoStar Omega, BMG Labtech). Readings were normalized to the signals measured in culture medium with resazurin alone. The assay was repeated at least three times for each condition ($n\ge 3$). Mean values and standard deviations are reported.

## Conclusions

Tumour spheroids provide an *in vitro* model that more closely mimics the physiological environment of tumours *in vivo* than do monolayer cultures. The main purpose of this study was to analyse the response of tumour spheroids to treatments of radiation, hyperthermia and combinations thereof and to test if biological equivalent dose calculations based on clonogenic cell survival provide a good measure of heat-induced radiosensitization. It was found that cells within tumour spheroids are more heat resistant than those in 2D culture and that spheroids may proliferate at a faster rate following heating. Moreover, we identified that the spheroid growth dynamics are significantly influenced by the treatment modality and suggest that this is due to the (in-) dependence of cell death on cell proliferation. This may cause changes of the cellular microenvironment, such as re-oxygenation of more central cell layers following hyperthermia treatment. Both, the microenvironmental changes and the increase in spheroid proliferation induced by hyperthermia warrant possible benefits for following fractionated radiation treatments. Further experimental validation of this hypothesis, as well as study of more physiological tumour models, such as tumours *in vivo*, or spheroid co-cultures of different (non-) malignant cell types, are needed to provide a full picture to quantify the radiosensitization potential of hyperthermia treatments. Finally, different treatment strategies also accounting for variations in heating temperature, sequence and time between the irradiation and heating, as well as fractionation need to be conducted. We can, however, conclude based on our preliminary study that radiation dose weighting based on clonogenic cell survival analysis alone may be insufficient to predict treatment efficacy of combination treatments since spheroid growth after radiation alone, hyperthermia alone or combined radiation-hyperthermia at the same BEQDs, differed significantly. Thus, more sophisticated biological dose concepts, potentially based on spheroid data, need to be suggested.

## Supplementary information

Video 1.

Video 2.

Video 3.

Video 4.

Supplementary Material.

## Data Availability

The data sets used and/or analysed during the current study are available from the corresponding author on reasonable request.

## References

[1] Pampaloni F, Reynaud EG, Stelzer EHK (2007). The third dimension bridges the gap between cell culture and live tissue. Nat. Rev. Mol. Cell Biol..

[2] Vinci M (2012). Advances in establishment and analysis of three- dimensional tumor spheroid-based functional assays for target validation and drug evaluation. BMC Biol..

[3] Sankar PS (2017). Modeling nasopharyngeal carcinoma in three dimensions (Review). Oncol. Lett..

[4] Edmondson Rasheena, Broglie Jessica Jenkins, Adcock Audrey F., Yang Liju (2014). Three-Dimensional Cell Culture Systems and Their Applications in Drug Discovery and Cell-Based Biosensors. ASSAY and Drug Development Technologies.

[5] Kadletz L (2015). Evaluation of spheroid head and neck squamous cell carcinoma cell models in comparison to monolayer cultures. Oncol. Lett..

[6] Dubessy C, Merlin JL, Marchal C, Guillemin F (2000). Spheroids in radiobiology and photodynamic therapy. Critical Rev. Oncol..

[7] Riedl A (2017). Comparison of cancer cells in 2D vs 3D culture reveals differences in AKT-mTOR-S6K signaling and drug responses. J. Cell Sci..

[8] Nath S, Devi GR (2016). Three-dimensional culture systems in cancer research: Focus on tumor spheroid model. Pharmacol. Ther..

[9] Riffle S, Pandey RN, Albert M, Hegde RS (2017). Linking hypoxia, DNA damage and proliferation in multicellular tumor spheroids. BMC Cancer.

[10] Paullin T (2017). Spheroid growth in ovarian cancer alters transcriptome responses for stress pathways and epigenetic responses. PLoS One.

[11] Jeppesen M (2017). Spheroid culture of primary colorectal cancer cells from liver metastases as an *in vitro* model of patient tumors. PLoS One.

[12] Reynolds DS (2017). Breast Cancer Spheroids Reveal a Differential Cancer Stem Cell Response to Chemotherapeutic Treatment. Sci. Reports.

[13] Durand RE (1978). Effects of hyperthermia on the cycling, noncycling, and hypoxic cells of irradiated and unirradiated multicell spheroids. Radiat. Res..

[14] Lücke-Huhle C, Dertinger H (1977). Kinetic Response of an *In Vitro* “Tumour-model” (V 79 spheroids) to 42 °C Hyperthermia. Eur. J. Cancer.

[15] Eynali S, Khoei S, Khoee S, Esmaelbeygi E (2017). Evaluation of the Cytotoxic Effects of Hyperthermia and 5-Fluorouracil Loaded Magnetic Nanoparticles on Human Colon Cancer Cell Line HT-29. Int. J. Hyperth..

[16] Rajaee Z, Khoei S, Rabi S, Marzieh M, Sakine E (2018). Evaluation of the effect of hyperthermia and electron radiation on prostate cancer stem cells. Radiat. Environ. Biophys..

[17] Song AS, Najjar AM, Diller KR (2014). Thermally Induced Apoptosis, Necrosis, and Heat Shock Protein Expression in 3D Culture. J. Biomech. Eng..

[18] Khoei S, Goliaei B, Neshasteh-riz ALI (2004). Differential thermo-resistance of multicellular tumor spehroids. Iran. J. Sci. Technol..

[19] Sutherland, R. M., McCredie, J. A. & Inch, W. R. Growth of multicell spheroids in tissue culture as a model of nodular carcinomas. *J*. *Natl*. *Cancer Inst*. **46**, 113–120, 10.1093/jnci/46.1.113, http://oup.prod.sis.lan/jnci/article-pdf/46/1/113/2712819/46-1-113.pdf (1971). 5101993

[20] Thomsen AR (2018). A deep conical agarose microwell array for adhesion independent three-dimensional cell culture and dynamic volume measurement. Royal Soc. Chem..

[21] Ivanov DP, Grabowska AM (2017). Spheroid arrays for high-throughput single-cell analysis of spatial patterns and biomarker expression in 3D. Sci. Reports.

[22] Li L, Zhou Q, Voss TC, Quick KL, Labarbera DV (2016). High-throughput imaging: Focusing in on drug discovery in 3D. Methods.

[23] Kunz-Schughart L, Freyer JP, Hofstaedter F, Ebner R (2004). The Use of 3-D Cultures for High-Throughput Screening: The Multicellular Spheroid Model. J. Biomol. Screen..

[24] Horsman MR, Overgaard J (2007). Hyperthermia: a Potent Enhancer of Radiotherapy. Clin. Oncol..

[25] Kampinga HH (2006). Cell biological effects of hyperthermia alone or combined with radiation or drugs: A short introduction to newcomers in the field. Int. J. Hyperth..

[26] Dikomey E, Jung H (1991). Thermal radiosensitization in CHO cells by prior heating at 41–46 °C. Int. J. Radiat. Biol..

[27] ter Haar G, Coussios C (2007). High intensity focused ultrasound: Physical principles and devices. Int. J. Hyperth..

[28] ter Haar G, Coussios C (2007). High intensity focused ultrasound: Past, present and future. Int. J. Hyperth..

[29] Martinho Costa, M. F. *Preclinical investigation of combined Focused Ultrasound and Radiotherapy to improve tumour response to treatment*. Ph.D. thesis, University of London (2017).

[30] van Leeuwen CM (2017). 3D radiobiological evaluation of combined radiotherapy and hyperthermia treatments. Int. J. Hyperth..

[31] Kok HP (2014). Quantifying the combined effect of radiation therapy and hyperthermia in terms of equivalent dose distributions. Int. J. Radiat. Oncol. Biol. Phys..

[32] Lauber K (2015). Targeting the heat shock response in combination with radiotherapy: Sensitizing cancer cells to irradiationinduced cell death and heating up their immunogenicity. Cancer Lett..

[33] Richter K, Haslbeck M, Buchner J (2010). The Heat Shock Response: Life on the Verge of Death. Mol. Cell.

[34] Lauber K, Ernst A, Orth M, Herrmann M, Belka C (2012). Dying cell clearance and its impact on the outcome of tumor radiotherapy. Front. Oncol..

[35] Brüningk SC (2018). A comprehensive model for heat-induced radio-sensitisation. Int. J. Hyperth..

[36] Durand RE, Sutherland RM (1972). Effects of intercellular contact on repair of radiation damage. Exp. Cell Res..

[37] Gao F, Ye Y, Zhang Y, Yang J (2013). Water bath hyperthermia reduces stemness of colon cancer cells. Clin. Biochem..

[38] Carlsson J, Acker H (1988). Relationships between pH, oxygen partial pressure and growth in cultured cell spheroids. Int. J. Cancer.

[39] Overgaard J (1976). Influence of extracellular ph on the viability and morphology of tumor cells exposed to hyperthermia. J. Natl. Cancer Inst..

[40] Freeman ML, Dewey WC (1977). Effect of pH on hyperthermic cell survival: Brief communication. J. Natl. Cancer Inst..

[41] Song, C. W., Lyons, J. C., Griffin, R. J., Makepeace, C. M. & Cragoe, E. J. Increase in Thermosensitivity of Tumor Cells by Lowering Intracellular pH. *Cancer Res*. **53**, 1599–1601, http://cancerres.aacrjournals.org/content/53/7/1599.full.pdf (1993). 8384080

[42] Kaida A, Miura M (2013). Visualizing the effect of tumor microenvironments on radiation-induced cell kinetics in multicellular spheroids consisting of HeLa cells. Biochem. Biophys. Res. Commun..

[43] Onozato Y, Kaida A, Harada H, Miura M (2017). Radiosensitivity of quiescent and proliferating cells grown as multicellular tumor spheroids. Cancer Sci..

[44] Yi PN (1987). Swelling of multicellular spheroids induced by hyperthermia. Int. J. Hyperth..

[45] Harmon BV (1990). Cell death induced in a murine mastocytoma by 42–47 °C heating *in vitro*: evidence that the form of death changes from apoptosis to necrosis above a critical heat load. Int. J. Radiat. Biol..

[46] Zhang Hua-Gang, Mehta Keyur, Cohen Patrice, Guha Chandan (2008). Hyperthermia on immune regulation: A temperature’s story. Cancer Letters.

[47] Yagawa Y, Tanigawa K, Kobayashi Y, Yamamoto M (2017). Cancer immunity and therapy using hyperthermia with immunotherapy, radiotherapy, chemotherapy, and surgery. J. Cancer Metastasis Treat..

[48] Crezee J (2016). Biological modelling of the radiation dose escalation effect of regional hyperthermia in cervical cancer. Radiat. Oncol..

[49] Franken NAP (2013). Cell survival and radiosensitisation: Modulation of the linear and quadratic parameters of the LQ model (Review). Int. J. Oncol..

[50] Jordan BF, Sonveaux P (2012). Targeting tumor perfusion and oxygenation to improve the outcome of anticancer therapy. Front. Pharmacol..

[51] Gerweck LE, Gillette EL, Dewey WC (1974). Killing of Chinese hamster cells *in vitro* by heating under hypoxic or aerobic conditions. Eur. J. Cancer.

[52] Gerweck, L. E., Nygaard, T. G. & Burlett, M. Response of cells to hyperthermia under acute and chronic hypoxic conditions. *Cancer Res*. **39**, 966–972, http://cancerres.aacrjournals.org/content/39/3/966.full.pdf (1979). 34477

[53] Rao W, Deng Z-S, Liu J (2010). A Review of Hyperthermia Combined With Radiotherapy/Chemotherapy on Malignant Tumors. Critical Rev. Biomed. Eng..

[54] Wust P (2002). Review Hyperthermia in combined treatment of cancer. The Lancet-Oncology.

[55] Mallory M, Gogineni E, Jones GC, Greer L, Simone CB (2016). Therapeutic hyperthermia: The old, the new, and the upcoming. Critical Rev. Oncol..

[56] Borrelli, M. J., Thompson, L. L., Cain, C. A. & Dewey, W. C. Time-temperature analysis of cell killing of BHK cells heated at temperatures in the range of 43.5 degrees C to 57.0 degrees C. *Int*. *J*. *Radiat*. *Oncol*. *Biol*. *Phys*. **19**, 389–399, https://doi.org/0360-3016(90)90548-X[pii] (1990). 10.1016/0360-3016(90)90548-x 2394618

[57] Thompson SM (2014). Heat stress induced cell death mechanisms in hepatocytes and hepatocellular carcinoma: *In vitro* and *in vivo* study. Lasers Surg. Medicine.

[58] Sapareto SA, Dewey WC (1984). Thermal dose determination in cancer therapy. Int. J. Radiat. Oncol. Biol. Phys..

[59] Kessel S (2017). Real-Time Viability and Apoptosis Kinetic Detection Method of 3D Multicellular Tumor Spheroids Using the Celigo Image Cytometer. J. Int. Soc. Adv. Cytom. Part A.

[60] Mulholland T (2018). Drug screening of biopsy-derived spheroids using a self-generated microfluidic concentration gradient. Sci. Reports.

[61] Cribbes S, Kessel S, McMenemy S, Qiu J, Chan LLY (2017). A Novel Multiparametric Drug-Scoring Method for High-Throughput Screening of 3D Multicellular Tumor Spheroids Using the Celigo Image Cytometer. SLAS Discov..

[62] Navab Nassir, Hornegger Joachim, Wells William M., Frangi Alejandro F. (2015). Medical Image Computing and Computer-Assisted Intervention – MICCAI 2015.

