# Supplementary material for: 3D tumour spheroids for the prediction of the effects of radiation and hyperthermia treatments
Source: Sci Rep. 2020 Feb 3;10:1653. doi: 10.1038/s41598-020-58569-4 (PMC6997397; doi:10.1038/s41598-020-58569-4)
Supplement: Supplementary file 5 — Supplementary Material. [file 41598_2020_58569_MOESM5_ESM.pdf]

3D tumour spheroids for the prediction of the effects of radiation and hyperthermia treatments

Sarah C. Brüningk[1*], Ian Rivens[1], Carol Box[1], Uwe Oelfke[1], and Gail ter Haar[1]

[1] *Joint Department of Physics at The Institute of Cancer Research and The Royal Marsden NHS Foundation Trust, London, SM25NG, UK*

[*] *sarah.brueningk@icr.ac.uk*

Supplementary material

Method used for histological analysis

For histological analysis, 6-8 spheroids per condition were incubated with pimonidazole hydrochloride (Hypoxyprobe™, Burlington, USA) at a concentration of 0.2 mM overnight. Spheroids were collected into 1.5 ml Eppendorf tubes, washed in phosphate buffered saline, and fixed overnight in 4% paraformaldehyde solution (Fisher Scientific Inc., Loughborough, UK) at room temperature. Fixed spheroids were embedded in 60°C 4% w/v agarose (ThermoFisher Scientific, East Grinstead, UK) gel. Once solidified, agarose blocks were transferred into tissue processing cassettes and processed in an automated Tissue-Tek vacuum infiltration processor (Sakura Finetek Europe, Alphen aan den Rijn, Netherlands) before embedding in paraffin. Sections (3µm thick) were cut and mounted on microscopy slides (Superfrost, VWR Int., Lutterworth, UK). Sections were dewaxed, rehydrated and subjected to antigen unmasking in 95°C, 10 mM sodium citrate (pH 6) for 50 min. Cooled slides were permeabilized in 0.025% Triton X-100 in Tris buffered saline for 2 x 3 min and blocked for 1 h in Tris buffered saline with 1% goat serum (Sigma Aldrich Ltd., Dorset, UK), and 10% bovine serum albumin (Sigma Aldrich Ltd.). Immunofluorescent co-staining for pimonidazole adducts and Ki-67 was performed as follows: overnight incubation at 4°C with anti-Ki-67 antibody (ab16667, Abcam, Cambridge, UK) at concentration 1:100 in blocking buffer, followed by washing in Tris buffered saline (3 x 5 min) before 1.5 h incubation with secondary antibody (goat anti-rabbit IgG, Alexa Fluor 555, A21428, ThermoFisher Scientific) at concentration 1:400 in blocking buffer at room temperature in the dark. After washing (3 x 5 min in Tris buffered saline) slides were incubated for 2 h with anti-pimonidazole antibody (Hypoxyprobe™ MAb kit, Hypoxyprobe) at concentration 1:200 in blocking buffer at room temperature in the dark. DAPI (D9542, Sigma Aldrich Ltd.) was used as a DNA stain at a concentration of 1:1000, incubated for 10 min. Slides were mounted in antifade mounting medium (Vector Laboratories, UK) and imaged on a Zeiss LSM700 confocal laser scanning microscope (Zeiss, Oberkochen, Germany). Alternatively tissues were stained with haematoxylin and eosin before being mounted in DPX mountant (VWR Int.). In this case imaging was performed on an optical microscope (Axio Scan, Zeiss, Oberkochen, Germany).

Supplementary figures

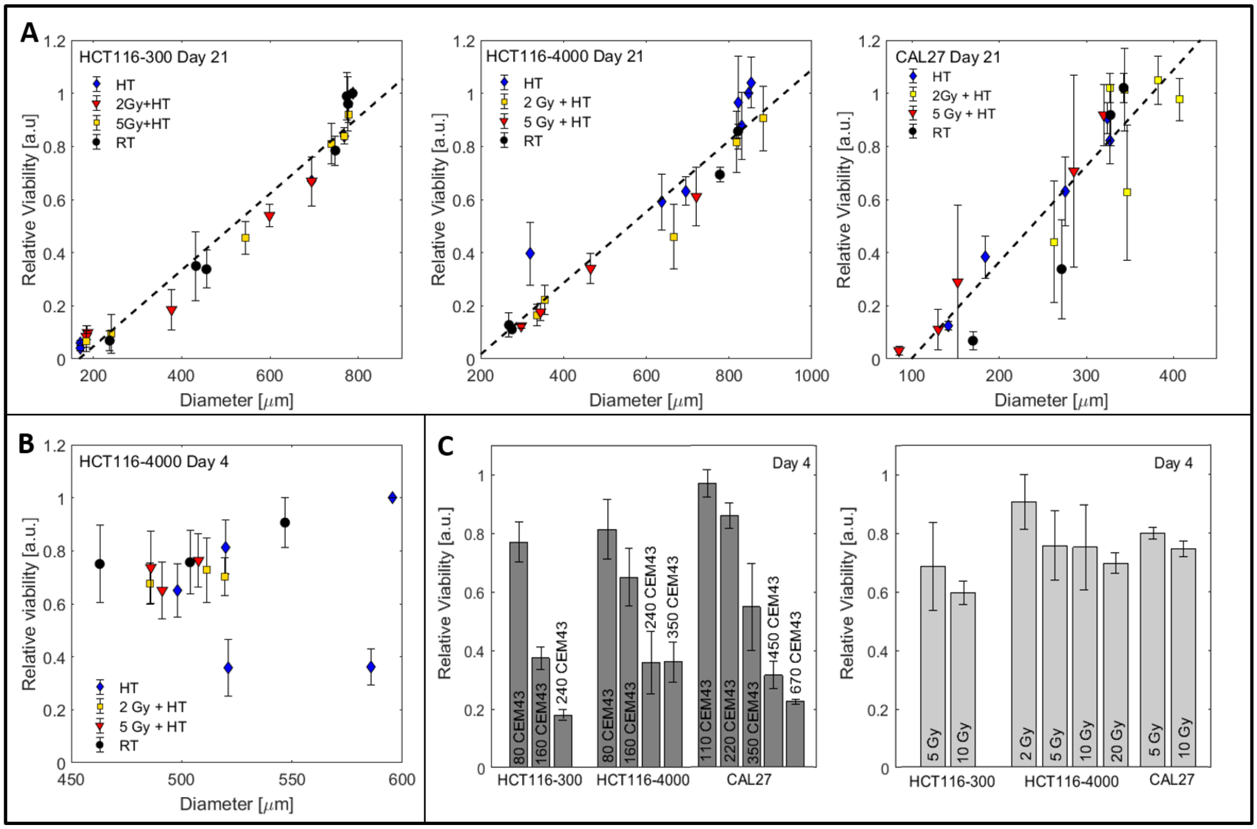

Figure S1: Cell viability measurements within spheroids. **A, B**: Viability of cells in treated, relative to those in untreated (control) spheroids. Mean values and standard deviations of viability measurements on day 21 (A - HCT116-300, HCT116-4000, CAL27) and day 4 (B - HCT116-4000) are plotted as a function of spheroid diameter. Dashed lines indicate linear fits of the data. Three weeks post treatment, spheroid diameter correlated linearly with cell viability (coefficient of determination R^2=0.98 (HCT116-300), R^2=0.92 (HCT116-4000), R^2=0.86 (CAL27)). This was not the case four days after treatment. **C**: Mean values and standard deviations of viability measurements on day 4 for HT (left) and RT (right) treatments at different doses. Viability decreased with thermal dose, but there was no statistically significant difference between viability of irradiated samples.

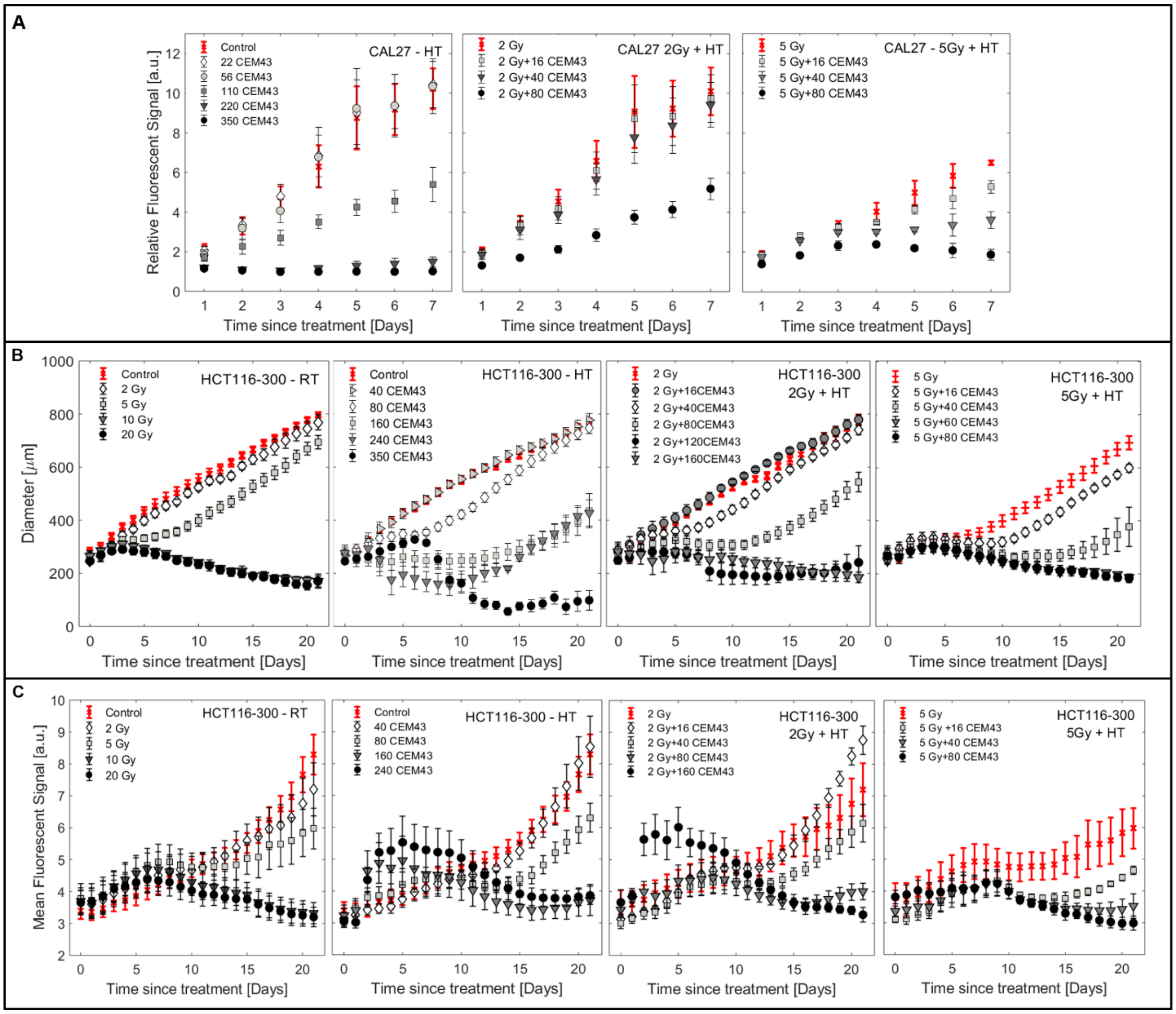

Figure S2: Additional 2D and 3D growth curves. **A**: Monolayer growth curves for 2000 CAL27 cells measured by the resazurin assay for a subset of treatments shown in figures 1A, 3A, and 4A. **B**: Growth curves of HCT116-300 spheroids following treatment. Spheroids were either irradiated (left), heated (2nd from left), or treated with a combination of either 2 Gy (3rd from left), or 5 Gy (right) with HT. **C**: Dynamic variation in mean PI staining intensity as a function of time since seeding in HCT116-300 spheroids. In all graphs (A, B and C) means and standard errors of the mean of at least three independent repeat experiments are shown (n≥3).

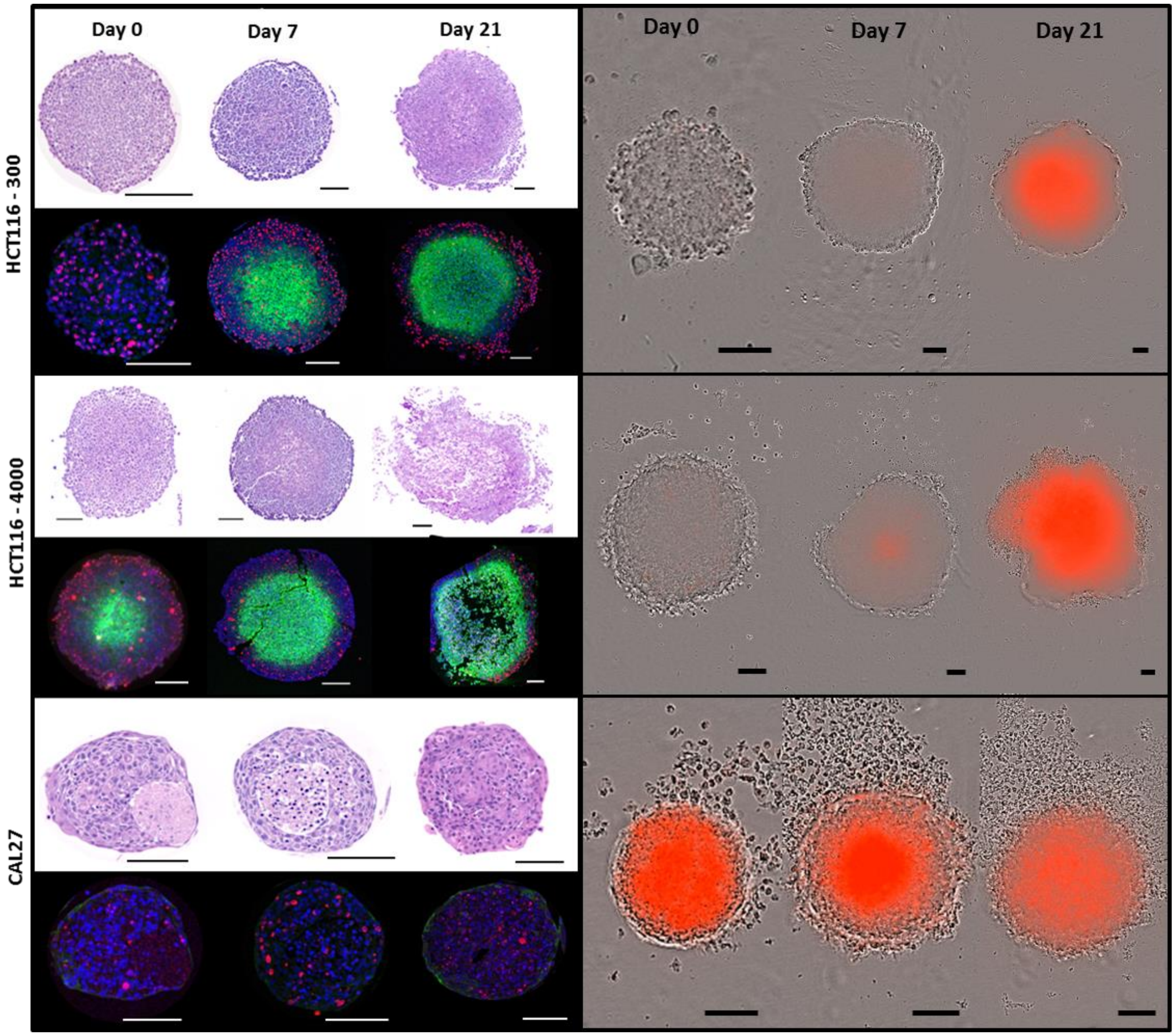

Figure S3: Morphology of untreated spheroids. Histological sections (left) and phase contrast overlaid with PI fluorescence time-lapse imaging snap shots (right) of untreated HCT116-300, HCT116-4000, and CAL27 spheroids obtained at different days after the 96 h formation period. Sections were stained with either haematoxylin and eosin (top rows) or using fluorescence labelling: DAPI (blue, nuclei), pimonidazole (green, hypoxia), and Ki-67 (red, proliferating cells). Scale bars are 100 µm. HCT116 spheroids formed from either 300 or 4000 cells both showed no signs of necrosis on day 0. HCT116-4000 spheroids displayed a hypoxic, yet not necrotic core at this time point. With increasing spheroid diameter the hypoxic region within HCT116 spheroids increased and a distinct layered structure of proliferating rim, hypoxic shell and necrotic core established. CAL27 spheroids displayed distinct necrotic patches on day 0, but on day 7 and over the observation period no significant pimonidazole staining, indicating a lack of hypoxia, was observed.

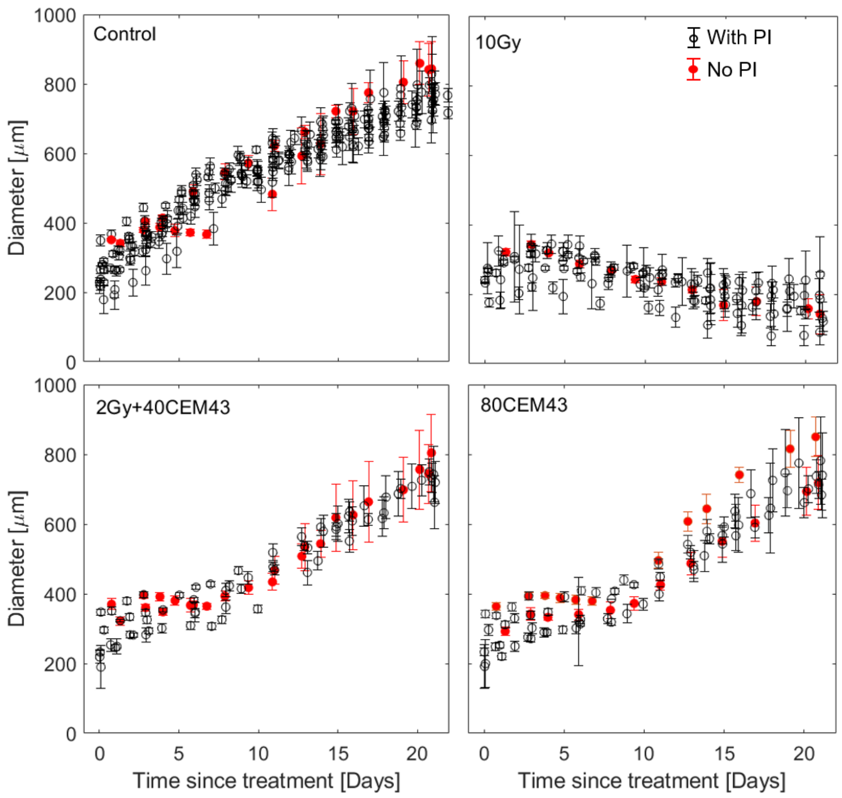

Figure S4: Influence of PI on spheroid growth. Growth curves of HCT116-300 spheroids, both control and treated, with (black, open circles) or without (red, filled circles) PI added at a concentration of 5 µg/ml for 21 days. Each data point represents the mean and standard deviation of three to six individual spheroids at a given time point. Data was not averaged over independent experimental repeats since n<3 for measurements without PI. There was no significant difference in spheroid diameter when PI was present in the culture medium in either control, irradiated, heated or combination treatments.
